# Alternative method for assessment of southwestern Atlantic humpback whale population status

**DOI:** 10.1371/journal.pone.0259541

**Published:** 2021-11-17

**Authors:** Guilherme A. Bortolotto, Len Thomas, Philip Hammond, Alexandre N. Zerbini

**Affiliations:** 1 Sea Mammal Research Unit, Scottish Oceans Institute, University of St Andrews, St Andrews, Fife, United Kingdom; 2 Centre for Research into Ecological and Environment Modelling, University of St Andrews, St Andrews, Fife, United Kingdom; 3 Instituto Aqualie, Juiz de Fora, Minas Gerais, Brazil; 4 Cooperative Institute for Climate, Ocean and Ecosystem Studies, University of Washington and Marine Mammal Laboratory, Alaska Fisheries Science Center, National Marine Fisheries Service, National Oceanic and Atmospheric Administration, Seattle, Washington State, United States of America; 5 Marine Ecology and Telemetry Research, Seabeck, Washington State, United States of America; Senckenberg am Meer Deutsches Zentrum fur Marine Biodiversitatsforschung, GERMANY

## Abstract

The population of humpback whales (*Megaptera novaeangliae*) wintering off eastern South America was exploited by commercial whaling almost to the point of extinction in the mid-twentieth century. Since cessation of whaling in the 1970s it is recovering, but the timing and level of recovery is uncertain. We implemented a Bayesian population dynamics model describing the population’s trajectory from 1901 and projecting it to 2040 to revise a previous population status assessment that used Sampling-Importance-Resampling in a Bayesian framework. Using our alternative method for model fitting (Markov chain Monte Carlo), which is more widely accessible to ecologists, we replicate a “base case scenario” to verify the effect on model results, and introduce additional data to update the status assessment. Our approach allowed us to widen the previous informative prior on carrying capacity to better reflect scientific uncertainty around historical population levels. The updated model provided more precise estimates for population sizes over the period considered (1901–2040) and suggests that carrying capacity (*K*: median 22,882, mean 22,948, 95% credible interval [CI] 22,711–23,545) and minimum population size (*N*_*1958*_: median 305, mean 319, 95% CI 271–444) might be lower than previously estimated (*K*: median 24,558, mean 25,110, 95% CI 22,791–31,118; *N*_*1958*_: median 503, mean 850, 95% CI 159–3,943). However, posterior 95% credible intervals of parameters in the updated model overlap those of the previous study. Our approach provides an accessible framework for investigating the status of depleted animal populations for which information is available on historical mortality (e.g., catches) and intermittent estimates of population size and/or trend.

## Introduction

Investigating the consequences and level of impact of wildlife exploitation is important to understand the conservation status of animal species, to evaluate the need for and guide implementation of management actions. Whaling during the twentieth century may be considered the greatest human exploitation of wildlife in history [1]. In the Southern Hemisphere, modern whaling reduced most humpback whale populations to small fractions of their pre-exploitation sizes [2]. The population breeding in Brazilian waters, the western south Atlantic (WSA) population, is no exception and, despite international protection regulations established in 1966, Soviet catches in the late 1960s and early 1970s [3] contributed to keep the population at depleted levels [4].

Humpback whales in the WSA population spend the austral winter and spring months in the coastal waters of Brazil, where pregnant females give birth and mating occurs [5, 6]. These animals migrate every year between wintering grounds in Brazil and feeding habitats around South Georgia and the South Sandwich Islands [7, 8]. It is well documented that the population size has increased greatly in the last 20 years [4, 6, 9–12]. Using a continuous exponential growth model fitted to abundance estimates derived from aerial line-transect surveys, Wedekin et al. [12] estimated that this population has grown extremely fast for cetaceans with similar life histories, at 12% (95% CI = 8-16%; CV = 0.17) per year from 2002 to 2011. The increase in whale numbers coincides with an increase in human activities in their area of distribution, such as gas and oil extraction activities [10, 13]. Conservation concern arises from potential impacts from such activities (e.g., from increased noise, vessel traffic, and chemical pollution) overlapping whale distribution.

It has been estimated that around 20,000 humpback whales were present in the Brazilian breeding grounds in 2012 (N = 20,389, CV = 0.071; [10]). This figure represents 80% of what was estimated in Zerbini et al. [4] to be the population size before 1900, when commercial whaling in the area started to adopt modern methods such as explosive harpoons and steam-powered boats [14]. To assess the population’s conservation status and its size before exploitation (i.e., pre-1900; assuming the population was at carrying capacity), that study [4] presented a model of population trajectory fitted to a 2005 abundance estimate from aerial survey data [6]. That aerial survey estimate is likely biased low due to visibility bias [9]. New information is available (i.e., abundance and growth rate estimates) and therefore the assessment of this population’s conservation status would benefit from re-evaluation using this new information.

In earlier efforts to assess the conservation status of humpback whales in the WSA [15], the International Whaling Commission Scientific Committee (IWC-SC), a group formed mainly by researchers who provide scientific advice to the IWC, had proposed different scenarios for catch allocation to account for uncertainty about mixing of breeding stocks on feeding grounds [16, 24]. That uncertainty arises from the possibility of whales caught in WSA migration, breeding, or feeding grounds, which might not from the WSA population. Information from satellite tracking and individual animal identification [4, 7, 8, 16] support that waters near South Georgia and South Sandwich Islands represent their main feeding grounds.

Population dynamics models can be used to describe the trajectory of population size over time. To calibrate such models using biological parameters that have uncertainty associated with them (e.g., estimated abundances, with confidence intervals or standard error), statistical tools that account for uncertainty are required to derive robust results. State Space Models (SSM) are an appropriate statistical framework for this [17–19]. A SSM contains two linked components: a “process model” that describes in our case how the true but unknown population sizes change over time and an “observation model” that describes how the true population sizes are linked to the observations. Both components have model parameters: the process model parameters govern the population dynamics while the observation model parameters describe any bias and uncertainty in the observations. SSMs are often implemented in a Bayesian framework, where informative prior distributions can be specified for the model parameters, reflecting biological knowledge on their possible values [17–19].

To assess the conservation status of WSA humpback whales, Zerbini et al. [4] used a Bayesian SSM to estimate the population trajectory and parameters in a density-dependent, sex and age-aggregated population dynamics model [20]. (We note that, strictly speaking, their model was not an SSM because the population dynamics model was deterministic, while in an SSM both the process and observation models are stochastic). That model was fitted using a numerical technique called Sampling Importance Resampling (SIR; e.g., [21]). A backward-projection approach [22] was used to estimate the population size in 1901, projecting back in time from a relatively recent abundance estimate for 2005 [6]. Population size in 1901 was considered to represent the population carrying capacity (*K*), assuming it was not depleted by unnatural deaths before that. However, small scale whaling in the area before that year [23], may have previously depleted the population, meaning that the population may not have been at carrying capacity at that time.

Here, the assessment of the conservation status of WSA humpback whale population was revisited by implementing and updating a previously published population dynamics model [4], but using a different method for model fitting. Objectives here were: to validate the new fitting method by reproducing the results described in Zerbini et al. [4]; to investigate the effect of including additional data (i.e., population abundance [10] and growth information [12]); to investigate model structure, including the effect of prior distributions and data inclusion on model outputs. An alternative Bayesian method to fit that model in a state-space framework was used, with which a wider range of researchers will be familiar: Markov chain Monte Carlo (MCMC) using the Metropolis algorithm [24]. We draw attention to the effects of having a very informative prior on pre-exploitation population size (*K*) and investigate widening it. Our results can contribute to the understanding of the impact that modern whaling had on the WSA humpback whale population and inform the need for management actions.

## Materials and methods

The population dynamics model presented here was based on the “base case scenario” described in Zerbini et al. [4]. This base case scenario assumes that the WSA humpback whale population was at carrying capacity at the beginning of the 20^th^ century, before the onset of modern whaling activities. Custom functions were created in software R (version 3.4.2; [25]) to implement two models: 1) the “*Base Case model*”, to replicate the base case scenario previously published; and 2) the “*Updated model*”, in which new data were used to update the *Base Case model*. The code for implementing both models is provided in S1 Appendix.

### Population model

The population dynamics model used in Zerbini et al. [4] is a density-dependent, sex and age-aggregated generalised logistic equation [20] that describes the population trajectory from 1901, and projecting it to 2040. The deterministic structure of the model is

Nt+1=Nt+Nt⋅rmax⋅[1−(NtK)z]−Ct

where *N*_*t*_ is the population size at year *t*, *r*_*max*_ is the maximum net recruitment rate, *K* is the carrying capacity, *z* is a shape parameter, *C*_*t*_ is catch at year *t*.

Parameter *z* was fixed at 2.39 [26], which determines that the relative population level at which maximum net recruitment occurs is 0.6 K.

The above-described population model structure was used in all model scenarios investigated here as the process component in the state-space framework. Quantities of interest derived included the population size at the beginning of the period considered (carrying capacity, *K*), the maximum depletion level (*N*_*min*_*/K*), which represents the lowest population level reached, and the current (year 2019) depletion level of the population (*N*_*current*_*/K*). Depletion levels were calculated by dividing estimated values of *N* by estimated values of *K*. The year in which the minimum population size (*N*_*min*_) occurred was that which had the smallest median value of *N*.

### Data

In the present *Base Case model*, the same data considered in the base case scenario of Zerbini et al. [4] were used: abundance estimate for 2005, estimated growth rate for 1995–1998 [27] and the “core hypothesis” catch series (S1 Appendix). In the *Updated model*, population abundance estimates for 2008 and 2012 [10], the previously used population growth rate information (i.e., for 1995–1998), a recently published growth rate estimate for 2002–2011 [12], and the previously used catch series were considered (Table 1). The abundance estimate derived from aerial survey data for 2005 [6] considered by Zerbini et al. [4] was not used in the *Updated model* because that estimate is not consistent with those from [10] and is likely biased low (see *Discussion*). Abundance and population growth rate estimates have associated errors; the observation component of the state-space model included error structures for these parameters (see *Observation model*). Catch data were assumed to be known, i.e., observed without error.

**Table 1:** Data used for modelling population dynamics in the *Base Case model* and the *Updated model*. The “core hypothesis” catch series is included in the code in S1 Appendix.

Information	Data	Source	Base case	Updated
Abundance 2005	6,251 (CV = 0.17)	[6]	*	
Abundance 2008	14,264 (CV = 0.084)	[10]		*
Abundance 2012	20,389 (CV = 0.071)	[10]		*
Growth rate (*r*) for 1995–1998	0.074 (CV = 0.446)	[27]	*	*
Growth rate (*r*) for 2002–2011	0.1135 (CV = 0.115)	[12]		*
Catch series	“Core hypothesis”	[4]	*	*

The catch series used here represents numbers of whales killed during modern whaling activities, defined as the “core hypothesis” [28], reviewed and presented in Zerbini et al. [4]. In this core hypothesis, humpback whales killed at feeding grounds between longitudes 70-20ºW and latitudes 40-50ºS, plus those killed between 50-20ºW and to the south of 50ºS, excluding Falkland catches, are assumed to have belonged to the population breeding along the Brazilian coast. Whaling records from catcher boats operating from whaling stations in Brazil [29], and records of catches made by a Soviet pelagic fleet on Abrolhos Bank and in offshore areas along the central coast of South America, including illegal catches [3, 30], represent breeding ground catches.

### Sensitivity analysis

To investigate potential sources of differences from the *Base Case model* to the *Updated model*, five additional models (“Sensitivity analysis models”, SA) were run:

- SA1: the *Base Case model* was updated only with the additional information on population growth rate from [12];
- SA2: the *Base Case model* was updated by replacing the abundance estimate for 2005 [31] by the abundance estimate for 2008 [10];
- SA3: the *Base Case model* was updated by replacing the abundance estimate for 2005 by the abundance estimate for 2012 [10];
- SA4: the *Base Case model* was updated by replacing the abundance estimate for 2005 by abundance estimates for both 2008 and 2012 [10];
- SA5: the *Base Case model* was updated by not including any abundance or population growth rate data.

### Observation model

In the observation component of the state-space model it was assumed that each observation of population growth rate ($\hat{r}$) between any two years (*x* and *y*) followed a normal distribution:

$${\hat{r}}_{x,y}∼Normal(r_{x,y},\sigma_{{\hat{r}}_{x,y}}^{2})$$

where

$$r_{x,y}=\frac{\ln(N_{y})-\ln(N_{x})}{y-x}$$

and $\sigma_{{\hat{r}}_{x,y}}^{2}$, the observation variance, is assumed known (see *growth rate CVs* in Table 1; $\sigma_{{\hat{r}}_{x,y}}^{2}={\left({\hat{r}}_{x,y}\times \text{CV}\right)}^{2}$).

A lognormal distribution was assumed for observed abundance in year *t* $\left({\hat{N}}_{t}\right)$:

$${\hat{N}}_{t}∼Lognormal(\ln(N_{t}),\sigma^{2}{}_{log{\hat{N}}_{t}})$$

where the observation variance $\sigma^{2}{}_{log{\hat{N}}_{t}}$ is also assumed known.

### Prior distributions

The same prior distributions for the model parameters as used in the base case scenario of Zerbini et al. [4] were used here: a uniform distribution for the prior on population size in 2005 (*N*_*2005*_), with lower bound 500 and upper bound 22,000; a uniform distribution for the prior on *r*_*max*_ with lower bound zero, because negative values for maximum recruitment rate are biologically unreasonable, and an upper bound 0.106, which corresponds to the maximum reasonable value for growth rate for the species [32]. This upper bound was used here to replicate Zerbini et al. [4].

The population model and prior distributions on *N*_*2005*_ and *r*_*max*_ together implied a prior distribution for population size in the first year (*N*_*1901*_), i.e., the carrying capacity, *K*. A Monte Carlo sampling approach was used to derive this prior: a set of 10,000 independent samples were simulated from the prior distributions on *N*_*2005*_ and *r*_*max*_; for each sample, a univariate optimization was conducted using a bisection algorithm to find the value of *N*_*1901*_ that, when projected forward using the population model with the known catches and given value of *r*_*max*_, produced the given value of *N*_*2005*_. This approach was also used by Zerbini et al. [4] as part of their fitting algorithm, and is referred to by them as “backward projection”.

### Computation

MCMC was used to produce samples from the posterior distribution for the unknown parameters *r*_*max*_ and *N*_*2005*_; values for the other quantities of interest (e.g., population size in other years, and carrying capacity *K*) were obtained as part of the MCMC computation. To create the Markov chains, a simple Metropolis algorithm (see Appendix C in [24] for a tutorial) was implemented, employing a bivariate normal proposal centred on the current value of the unknown parameters. The bivariate proposal was used to allow flexibility to improve performance of the algorithm by allowing correlated proposals for *r*_*max*_ and *N*_*2005*_ if the posterior distributions were strongly correlated. The proposal variance for each parameter was set using trial and error, to keep the Metropolis acceptance ratio within the range 0.2–0.4 [24].

Simulating a large number of samples to estimate posterior distributions was facilitated by the relatively fast computation. The initial 20,000 samples were discarded as “burn-in”, and then 500,000 samples were simulated. To save on storage, and because successive samples were highly correlated, we thinned by keeping every 50th sample, which meant 10,000 samples were retained for inference. Three parallel chains (with differing start values) were simulated, to help assess chain convergence (using trace plots and Brooks-Gelman-Rubin statistics [33]; S2 Appendix); these chains were combined for inference and so a total of 30,000 samples were used as a sample from the posterior distribution.

Starting values for *N*_*2005*_ and *r*_*max*_ for each chain were chosen to be far enough apart to minimize the possibility of the Metropolis algorithm sampler misidentifying the most likely posterior distribution, i.e., preferentially sampling from a local maximum. Initial values for the three chains were 4,250, 6,250 and 8,250 for *N*_*2005*_, and 0.01, 0.07 and 0.100 for *r*_*max*_.

## Results

### Base Case model

**Fig 1:**
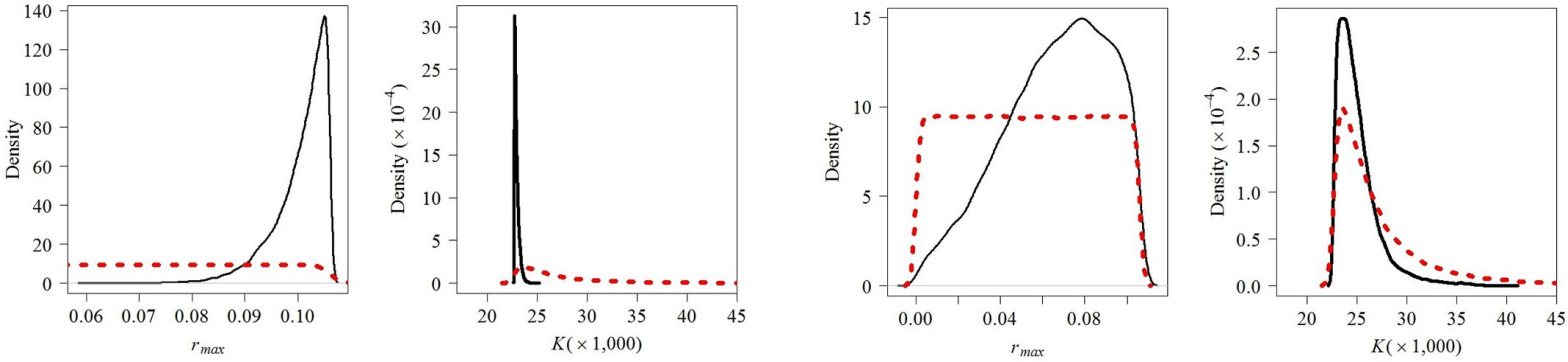
Posterior (continuous black lines) and prior (dashed red lines) distributions for *rmax* and *K* in the *Base Case model* (upper panel) and the *Updated model* (lower panel). Compare this figure with the base case scenario in [4].

**Fig 2:**
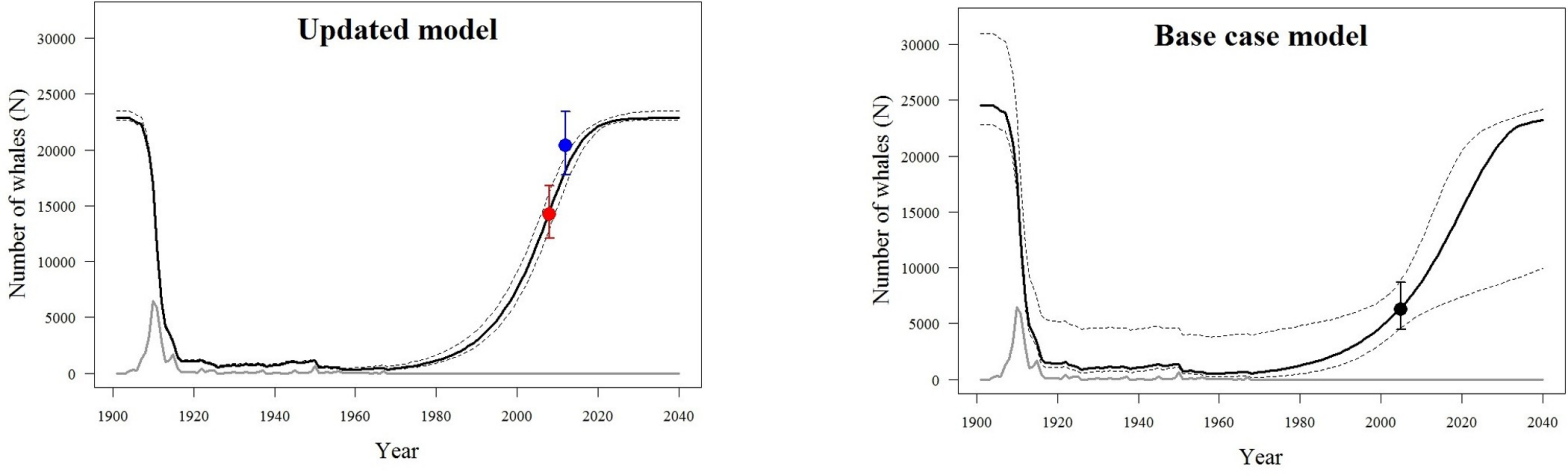
Population trajectories fitted for the *Base Case model* (upper) and the *Updated model* (lower). Black lines represent the posterior median (solid line) and 95% credible interval limits (dashed lines), estimated for population size. Observed abundance in 2005 [6], in 2008 and 2012 [10] are indicated, respectively, by black, red and blue filled circles with error bars (95% confidence interval). The grey line represents catches. Compare this figure with Zerbini et al. [4] *Base Case scenario*.

The *Base Case model* implemented here produced very similar posteriors and population trajectory to those presented in the previously published base case scenario (Table 2; compare Figs 1 and 2 with Fig 3 in [4]; Fig 3 from [4] is reproduced in Fig B in S3 Appendix). The present *Base Case model* suggested that the current depletion of the WSA humpback whale population is 60% (*N*_*2019*_: median = 14,552, 95% Credible Interval, CI = 7,282–19,874) of its carrying capacity (*K*: median = 24,524, 95% CI = 22,805–30,970). Trace plots excluding burn-in samples indicated model convergence (Fig A in S2 Appendix).

**Table 2:** Posterior medians, means and, lower (2.5%) and upper (97.5%) limits of 95% credible (equal-tailed) interval of model parameters in the base case scenario [4], the present *Base Case* and *Updated* models.

	***Base Case scenario*** [4]			
**Parameter**	**Median**	**Mean**	**2.5%**	**97.5%**
***r***_***max***_	0.069	0.066	0.013	0.104
***K***	24,558	25,110	22,791	31,118
***N***_***min***_	503	850	159	3,943
**Maximum depletion (year = 1958)**	0.020	0.031	0.007	0.125
	***Base Case model***			
	**Median**	**Mean**	**2.5%**	**97.5%**
***r***_***max***_	0.069	0.066	0.014	0.104
***K***	24,524	25,060	22,805	30,970
***N***_***min***_	496	843	233	3,809
**Maximum depletion (year = 1958)**	0.020	0.031	0.010	0.123
**Current depletion (year = 2019)**	0.597	0.581	0.240	0.860
	***Updated model***			
	**Median**	**Mean**	**2.5%**	**97.5%**
***r***_***max***_	0.102	0.101	0.088	0.106
***K***	22,882	22,948	22,711	23,545
***N***_***min***_	305	319	271	444
**Maximum depletion (year = 1958)**	0.013	0.014	0.012	0.019
**Current depletion (year = 2019)**	0.955	0.953	0.922	0.973

### Updated model

The *Updated model* produced different posterior distributions from the *Base Case model* (Fig 1), with carrying capacity estimated to be about two thousand animals fewer (*K*: median = 22,882, 95% CI = 22,711–23,545). Maximum recruitment rate was estimated to be about 50% higher (*r*_*max*_: median = 0.102, 95% CI = 0.088–0.106). The estimated maximum depletion median indicated the population size to have been as low as about 300 animals in 1958, almost 200 less than estimated in the *Base Case model*. However, credible interval limits for the minimum estimated population size in the *Updated model* (*N*_*min*_ 95% CI = 271–444) are completely within those from the *Base Case model* (*N*_*min*_ 95% CI = 233–3,809). Estimated depletion indicates that the population in 2019 was about 95% of its estimated carrying capacity (*N*_*2019*_: median = 21,878, 95% CI = 21,377–22,285), a larger population size estimate than indicated by the *Base Case model*. Estimated abundances describing population trajectory were also more precise, with narrower 95% credible intervals (Fig 2). The key piece of additional data causing this large increase in precision was further investigated in the *Sensitivity Analysis*. Similar to the *Base Case model*, trace plots again indicated model convergence (Fig B in S2 Appendix).

### Sensitivity analysis

Population trajectories estimated in the SA models (Fig A in S3 Appendix) indicate that SA1, which was the only SA scenario to consider growth rate in 2002–2011, had relatively more precise estimates for population sizes (Table in S3 Appendix). With the exception of this scenario and SA4, which included abundance estimates for both 2018 and 2012 (and excluded that for 2005), SA models had high uncertainty for *K*.

## Discussion

Here we successfully replicated the *Base Case scenario* presented in Zerbini et al. [4], using MCMC instead of SIR, and produced very similar outputs when the same combination of data was considered (i.e., the *Base Case model*). This illustrates that the SIR model implemented in that study can be replicated in a MCMC approach, providing independent verification of the model implementation. MCMC is a method with which ecologists are more familiar for estimating parameters in a Bayesian modelling framework [34], therefore the present implementation is more widely accessible to those researchers.

It is very clear from the population trajectory drawn for SA models (Fig A in S3 Appendix) that the growth rate information for 2002-2011 [12] is the main factor leading to improvement in precision (e.g., SA1). Growth rate for that nine-year period is relatively precise (CV = 0.115), and it represents a large portion of the data used for computing uncertainty (i.e., data observed with error). The model in SA4, which excluded the abundance data for 2005 [31] but included estimated abundances for 2008 and 2012 [10], which were also very precise ($\hat{N}$_*2008*_ CV = 0.084, $\hat{N}$_*2012*_ CV = 0.071) was the only other SA model to present an overall improvement in the population trajectory precision,. Overall, the new growth rate and abundance estimates considered in the *Updated model* seem to be consistent with each other. Another important point to highlight from the population trajectories (Fig A in S3 Appendix) and posterior estimates (Table in S3 Appendix) from the SA models, is that the posterior median for *K* was estimated to be very close to its prior in every scenario, although credible intervals are narrower the more data are considered. SA5, the scenario with no data considered other than the catch series, together with the above observations, show that the implied prior on *K* is very informative. That largely influences the posterior distribution for that parameter in the population assessment model on which the present study is based and also on updated population assessments (e.g., [35]).

With a detailed inspection of the model structure and specifications described in Zerbini et al. [4], some potential improvements become apparent. For example, the upper bound for the prior on *r*_*max*_ (= 0.106) was set based on a study that used a range of life history parameters from several humpback whale populations to compute the maximum plausible rate of population growth for the species [32]. However, a study that used additional data to what is presented in [32] suggested the maximum plausible growth rate could be slightly higher [= 0.118; 36]. Moreover, the estimates for *r*_*max*_ estimated from the *Updated model* indicated most of its posterior density to be close to the upper bound imposed (i.e., 0.106). Additionally, the value of growth rate between 2002-2011 used in the present *Updated model* [12] has a mean that is higher than that upper limit (*r*_*2002-2011*_ mean = 0.113, CV = 0.115), despite its 95% credible interval (*r*_*2002-2011*_ 95% CI = 0.088–0.139) including that value. An investigation into how to define a more appropriate prior on *r*_*max*_ should be considered in future modelling exercises to investigate population status.

A topic of future investigation relates to the prior on carrying capacity. The vague (i.e., not very informative) prior on *N*_*2005*_ combined with the vague prior on *r*_*max*_ leads to a very informative prior on *K* (Fig 1) given the structure of the population model and catch data, as implemented in Zerbini et al. [4]. For that reason, a vague prior on population size for any year in the base case would be expected to provide the same population trajectory and posteriors, so long as that prior was consistent with the implied value for *K* with the present prior on *N*_*2005*_. In this sense, a potentially better approach to model the trajectory for this population, and to estimate the parameters of interest, would be to set a very vague prior directly on *K*. This coupled with a better informed (i.e., not overly restricted) prior on *r*_*max*_ as described above, would likely provide important improvements on the present model implementation.

In the *Updated model*, maximum depletion was estimated to be even more severe than before [4], with the posterior 95% probability credible interval indicating that the population could have been depleted to as low as 1% of its carrying capacity. That represents a value close to the minimum plausible size for this population, suggested to be 264 [4]. This number was derived by applying a correction factor of four times [37] the number of mtDNA haplotypes found in whales from this population (= 66; [38]), assuming an even sex ratio, and likely provides a conservative minimum bound for the population [37]. Although results from the *Updated model* suggest that the median for the minimum population size was 305 (*Updated model N*_*min*_: median = 305, 95% CI = 271–444) in 1958, about 200 less than in the *Base Case model* (*Base Case model N*_*min*_: median = 496, 95% CI = 233–3,809), this number still agrees very well with the above conservative genetic constraint information. Moreover, since carrying capacity was estimated to be lower in the *Updated model*, with the posterior for maximum recruitment rate *r*_*max*_ indicating higher values to be more probable (*Updated model r*_*max*_: median = 0.102, 95% = 0.088–0.106) than in the *Base Case model* (*Base Case model r*_*max*_: median = 0.069, 95% = 0.014–0.104), the population trajectory curve indicates that the population could have reached carrying capacity around 2020 (Fig 2), sooner than indicated by Zerbini et al. [4] and predicted with the *Base Case model* (i.e., in around 2040).

In the modelling investigated here it is assumed that this population was at carrying capacity at the beginning of the period considered. Although there is information on pre-modern whaling catches, from before the 20^th^ century [23], there is substantial uncertainty in numbers caught for that period. One possibility is to include a minimum number of whales caught for years pre-1901, which would help investigating a possible minimum number for carrying capacity. The inclusion of this information would change model outputs [35], with carrying capacity necessarily larger. Investigating different scenarios for catch series can help to understand the sensitivity of the model to the uncertainty on which scenario is the most realistic [35]. Moreover, similarly to the observation processes described here for abundance and growth rate, a state-space model assuming that the catch data were observed with uncertainty can be fitted in a Bayesian framework. For that to be possible, measures of uncertainty for catch data are necessary, which currently do not exist. Also, the assumption that carrying capacity is constant over time may not be realistic, although this allows for a relatively simple modelling framework [39]. None of the models allow for the possibility of non-natural mortalities, other than those caused by whaling, which may currently occur [40, 41]. Information on the above points is sparse. For example, there are no estimates of recent mortality due to human impacts for this population. Exclusion of non-natural mortality unrelated to whaling for modelling the trajectory of this population likely leads to overestimation of the current depletion levels [35].

Because data inclusion was identified as a very important factor affecting model outputs [4], ways to include more information on population size should be considered in future investigations (e.g., [35]). For example, although the 2005 abundance estimate from Andriolo et al. [31] may be biased low, it could potentially be included in the *Updated model* if it were appropriately scaled up. That study used data collected in line transect aerial surveys to estimate WSA humpback whale population abundance and may have not adequately corrected for animals not detected on the trackline. Bortolotto et al. [9] discussed the differences in abundance estimates from line-transect ship survey data and from the line-transect aerial survey data used in [42] for the same year (i.e., 2008) and using similar survey team members, aircraft and survey protocol as [31]. If the difference between abundances presented in [9] and [42] can be used as a scaling factor to correct the abundance estimate from [31], the latter could possibly be included in the *Updated model* presented here. Additionally, similar to the different scenarios of data inclusion investigated in Zerbini et al. [4] other sources of estimated abundance could be considered to further update the present models (e.g., [11]).

Present results further support that the WSA humpback whale population is increasing and will recover to its size prior to modern whaling in the next few years. However, the *Updated model* resulted in a recovery scenario likely too optimistic. More realistic assessments of the conservation status of this population have considered information on pre-1901 whaling, leading to results that are different from those presented here. Future work should use a more complete set of input data ([35]; i.e., pre-modern whaling data, correction factors for “struck-and-lost” whales, and indices of abundance) to evaluate by how much model outputs could differ if the modelling approach implemented here study is used. The state-space population dynamics model implemented here using MCMC may be an accessible tool for ecologists to investigate the status of depleted populations.

## Supporting information

S1 Appendix Code for implementing the “Base case model” and the “Updated model”. (DOCX)

S2 Appendix Model convergence plots. (DOCX)

S3 Appendix Sensitivity analysis plots and table. (DOCX)
