# Supplementary material for: Alternative method for assessment of southwestern Atlantic humpback whale population status
Source: PLoS One. 2021 Nov 17;16(11):e0259541. doi: 10.1371/journal.pone.0259541 (PMC8598017; doi:10.1371/journal.pone.0259541)
Supplement: S1 Appendix — (DOCX) [file pone.0259541.s001.docx]

## S1 Appendix. Code for implementing the “Base case model” and the “Updated model”

##CODE FOR IMPLEMENTING THE BASE CASE MODEL

##Bayesian population model, MCMC used for fitting model based on “Zerbini et al. (2011) A Bayesian assessment of the conservation status of humpback whales in the WSA”

##Last update 20/Jan/2019 by GAB

##This version sets a prior on number of animals in 2005 and uses backward projection to derive an implied prior for number in the first year of inference (i.e.,1901)

library**(**parallel**)**

library**(**doParallel**)**

library**(**foreach**)**

library**(**doRNG**)**

#-----------------------------------------------------------------------------

# Function definitions

#-----------------------------------------------------------------------------

pop.trajectory **<-** **function(**rmax, K, catch, z **=** 2.39**){**

##Purpose: Create a population trajectory using the generalized logistic model of Zerbini et al. (2011)

# Inputs:

# rmax - max population growth rate

# K - carrying capacity (population assumed at carrying capacity at time 1)

# catch - vector of catches

# z - exponent in generalized logistic function

##Outputs:

# vector of population sizes, of length equal to length of catch vector

T **<-** length**(**catch**)**

n **<-** numeric**(**T**)**

n**[**1**]** **<-** K

**for(**t **in** 1**:(**T**-**1**)){**

# Equation 1 from Zerbini et al. (2011) paper

n**[**t **+** 1**]** **<-** n**[**t**]** **+** n**[**t**]** ***** rmax ***** **(**1 **-** **(**max**(**n**[**t**]**, 0**)** **/** K**)** **^** z**)** **-** catch**[**t**]**

**}**

return**(**n**)**

**}**

diff.k **<-** **function(**K, rmax, n.105, catch**)** **{**

##Purpose: Returns the difference between the projected population trajectory in year 105 (i.e., 2005) given K, rmax and catch and the "known" population value in that year, n.105. This function is called repeatedly by find.k which searches for the value of K that gives a diff closest to 0

n **<-** pop.trajectory**(**rmax, K, catch**)**

diff **<-** n**[**105**]** **-** n.105

return**(**diff**)**

**}**

find.K **<-** **function(**rmax, n.105, catch**){**

##Purpose: Searches for a value of K (population size in year 1) given values of rmax, n.105 ("known" number in 2005) and catch. Uses bisection algorithm implemented via uniroot function

tol **<-** 1E**-**3

res **<-** uniroot**(**diff.k, c**(**1, 100000**)**, tol **=** tol, rmax, n.105, catch**)**

return**(**res**$**root**)**

**}**

lnl **<-** **function(**n**)** **{**

##Purpose: return log-likelihood of pop trajectory n.

# Assumes (1) normal observation of growth rate in 1995-98 and (2) lognormal observation on population size in 2005

# Inputs:

# n - vector of population trajectory, starting in 1901

##Outputs:

# log-likelihood as a scalar

##Log growth rate 1995.1998

robs1995.1998 **<-** 0.074

sigma1995.1998 **<-** 0.033

##Population size in 2005

N2005 **<-** 6251

CV.N2005 **<-** 0.17

lN2005 **<-** log**(**N2005**)**

sigma.lN2005 **<-** sqrt**(**log**(**1 **+** CV.N2005 **^** 2**))**

##Calculate observed log growth rate

r1995.1998 **<-** **(**log**(**max**(**n**[**98**]**, 0**))** **-** log **(**max**(**n**[**95**]**, 1E**-**7**)))** **/** 3

##lnl of observed rate

res.1a **<-** **-(**log**(**sigma1995.1998**)** **+** 0.5 ***** **(**r1995.1998 **-** robs1995.1998**)** **^** 2 **/** sigma1995.1998 **^** 2**)**

##lnl of population size in 2005

res.2 **<-** **-(**log**(**sigma.lN2005**)** **+** lN2005 **+** 0.5 ***** **(**log**(**max**(**n**[**105**]**, 0**))** **-** lN2005**)** **^** 2 **/** sigma.lN2005 **^** 2**)**

res **<-** res.1a **+** res.2

**if** **(**is.na**(**res**))** res **<-** **-Inf**

return**(**res**)**

**}**

ln.prior **<-** **function(**rmax, K, n.105**)** **{**

##Purpose: returns log of prior on rmax, K and n in 2005

# Assume (1) uniform prior distribuion for rmax (0, 0.106)

# (2) uniform (500, 22000) prior on n in 2005

##Inputs:

# rmax - value of rmax

# n.105 - population size in 2005

# K - value of K

res.1 **<-** dunif**(**rmax, 0, 0.106, log **=** **TRUE)**

res.2 **<-** dunif**(**n.105, 500, 22000, log **=** **TRUE)**

res **<-** res.1 **+** res.2

**if(**is.na**(**res**))** res **<-** **-Inf**

return**(**res**)**

**}**

generate.samples**<-function(**B, thin, starting.values, catch,

proposal.sd.mult **=** c**(**0.05, 0.05**)**, proposal.rho **=** 0**){**

##Purpose: implements a random walk Metropolis sampler, based on a multivariate normal proposal

##Inputs:

# B - number of samples (before thinning)

# thin - number of samples for each 1 saved

# catch - vector of catches

# starting.values - starting values (list) $rmax and $n.105

# proposal.sd.mult - used for specifying the random walk sd - sd for each parameter (vector length 2)

# is initial value * proposal.sd.mult

# proposal.rho - correlation between proposal for rmax and K

##Outputs:

# rmax - vector of B/thin rmax values

# n.105 - vector of B/thin N2005 values

# K - vector for B/thin K values

# n - matrix (B/thin * length(catch)) abundance values

# p.accept - proportion of proposed values that were accepted

##Implementation note - requires the MASS library

# for multivariate normal distribution

require**(**MASS**)**

##Set up storage of samples

n.to.save **<-** B%/%thin

rmax **<-** n.105 **<-** K **<-** numeric**(**n.to.save**)**

n **<-** matrix**(**0, n.to.save, length**(**catch**))**

##Initialize chain

rmax.current **<-** rmax**[**1**]** **<-** starting.values**$**rmax

n.105.current **<-** n.105**[**1**]** **<-** starting.values**$**n.105

K.current **<-** K**[**1**]** **<-** find.K**(**rmax.current, n.105.current, catch**)**

n.current **<-** n**[**1, **]** **<-** pop.trajectory**(**rmax.current, K.current, catch**)**

ln.post **<-** lnl**(**n.current**)** **+** ln.prior**(**rmax.current, K.current, n.105.current**)**

##Random walk proposal specification

rmax.sigma.prop **<-** rmax.current ***** proposal.sd.mult **[**1**]**

n.105.sigma.prop **<-** n.105.current ***** proposal.sd.mult **[**2**]**

Sigma **<-** matrix**(**c**(**rmax.sigma.prop **^** 2,

rep**(**rmax.sigma.prop ***** n.105.sigma.prop ***** proposal.rho, 2**)**,

n.105.sigma.prop **^** 2**)**, 2, 2**)**

n.accept **<-** 0

**for** **(**i **in** 2**:**B**){**

##Propose new values

prop **<-** mvrnorm**(**1, c**(**rmax.current, n.105.current**)**, Sigma**)**

rmax.prop **<-** prop**[**1**]**

n.105.prop **<-** prop**[**2**]**

##Note: can't have zero or negative rmax or n.105

**if(**rmax.prop **>** 0 **&** n.105.prop **>** 0**)** **{**

K.prop **<-** find.K**(**rmax.prop, n.105.prop, catch**)**

n.prop **<-** pop.trajectory**(**rmax.prop, K.prop, catch**)**

ln.post.prop **<-** lnl**(**n.prop**)** **+** ln.prior**(**rmax.prop, K.prop, n.105.prop**)**

acceptance.prob **<-** min**(**1, exp**(**ln.post.prop **-** ln.post**))**

accept **<-** runif**(**1, 0, 1**)** **<** acceptance.prob

**if(**accept**)** **{**

n.accept **<-** n.accept **+** 1

rmax.current **<-** rmax.prop

n.105.current **<-** n.105.prop

K.current **<-** K.prop

n.current **<-** n.prop

ln.post **<-** ln.post.prop

**}**

**}**

##Save samples

**if** **((**i**-**1**)** %% thin **==** 0**)** **{**

s **<-** **(**i**-**1**)** %/% thin **+** 1

rmax**[**s**]** **<-** rmax.current

n.105**[**s**]** **<-** n.105.current

K**[**s**]** **<-** K.current

n**[**s, **]** **<-** n.current

**}**

**}**

p.accept **<-** n.accept **/** **(**B **-** 1**)**

return**(**list**(**rmax **=** rmax, n.105 **=** n.105, K **=** K, n **=** n, p.accept **=** p.accept**))**

**}**

#-----------------------------------------------------------------------------

# Inputs

#-----------------------------------------------------------------------------

##Catch data

##Base catches fom Zerbini et al., (2011) Table 4; Note: start in 1901

catch**<-**c**(**0, 0, 0, 180, 288, 240, 1261, 1849, 3391, 6468, 5832, 2881, 999,

1155, 1697, 447, 121, 129, 111, 102, 9, 364, 133, 266, 254, 7, 0,

19, 51, 107, 18, 23, 132, 57, 48, 105, 242, 0, 2, 36, 13, 0, 4, 60,

238, 30, 35, 48, 83, 698, 45, 34, 140, 44, 96, 167, 61, 16, 15, 27,

13, 24, 12, 0, 52, 0, 189, 0, 0, 0, 0, 2, 0, 0, 0, 0, 0, 0, 0, 0, 0,

0, 0, 0, 0, 0, 0, 0, 0, 0, 0, 0, 0, 0, 0, 0, 0, 0, 0, 0, 0, 0, 0, 0, 0**)**

##project forward to 2040

catch **<-** c**(**catch,numeric**(**35**))**

T **<-** length**(**catch**)**

years **<-** 1**:**T **+** 1900

##MCMC chain specifications

n.chains **<-** 3

B **<-** 5.2E5

thin **<-** 50

n.to.save **<-** B%/%thin

n.saved**<-**n.to.save ***** n.chains

burnin **<-** 4e2

n.retain.samples **<-** n.to.save**-**burnin

##Starting values

starting.values**<-**data.frame**(**

rmax **=** c**(**0.01, 0.07, 0.10**)**,

n.105 **=** c**(**4250, 6250, 8250**))**

##Comment this out to get a different result each time

set.seed**(**1234**)**

start.time **<-** Sys.time**()** #Record time to run

**if(**n.chains**>**1**)** **{**

##Run multiple chains in parallel

cl **<-** makePSOCKcluster**(**n.chains**)**

registerDoParallel**(**cl**)**

res **<-** foreach**(**chain **=** 1**:**n.chains**)** %dorng% **{**

generate.samples**(**B **=** B, thin **=** thin, starting.values **=** starting.values**[**chain,**]**, catch, proposal.sd.mult **=** c**(**0.5, 0.5**))**

**}**

stopCluster**(**cl**)**

**}** **else** **{**

##Run a single chain without using parallelization

res **<-** list**(**generate.samples**(**B **=** B, thin **=** thin, starting.values **=** starting.values**[**1,**]**, catch, proposal.sd.mult **=** c**(**0.5, 0.5**)))**

**}**

##Concatenate results from each chain

##Exclude burn-in samples: run only once, otherwise it will remove the burn-in amount of samples repeatedly

**for(**i **in** 1**:**n.chains**){**

res**[[**i**]]$**rmax **<-** res**[[**i**]]$**rmax**[(**burnin**+**1**):**length**(**res**[[**i**]]$**rmax**)]**

res**[[**i**]]$**n.105 **<-** res**[[**i**]]$**n.105**[(**burnin**+**1**):**length**(**res**[[**i**]]$**n.105**)]**

res**[[**i**]]$**K **<-** res**[[**i**]]$**K**[(**burnin**+**1**):**length**(**res**[[**i**]]$**K**)]**

res**[[**i**]]$**n **<-** res**[[**i**]]$**n**[(**burnin**+**1**):**nrow**(**res**[[**i**]]$**n**)**,**]**

**}**

str **<-** "c("

**for(**i **in** 1**:**n.chains**){**

str **<-** paste**(**str, "res[[", i, "]]$rmax", ifelse**(**i **<** n.chains, ", ", ")"**)**, sep**=**""**)**

**}**

rmax**<-**eval**(**parse**(**text**=**str**))**

str **<-** "c("

**for(**i **in** 1**:**n.chains**){**

str **<-** paste**(**str, "res[[", i, "]]$n.105", ifelse**(**i **<** n.chains, ", ", ")"**)**, sep**=**""**)**

**}**

n.105**<-**eval**(**parse**(**text**=**str**))**

str **<-** "c("

**for(**i **in** 1**:**n.chains**){**

str **<-** paste**(**str, "res[[", i, "]]$K", ifelse**(**i **<** n.chains, ", ", ")"**)**, sep**=**""**)**

**}**

K**<-**eval**(**parse**(**text**=**str**))**

str **<-** "rbind("

**for(**i **in** 1**:**n.chains**){**

str **<-** paste**(**str, "res[[", i, "]]$n", ifelse**(**i **<** n.chains, ", ", ")"**)**, sep**=**""**)**

**}**

n**<-**eval**(**parse**(**text**=**str**))**

n.median **<-** apply**(**n, 2, median**)**

n.lower **<-** apply**(**n, 2, quantile, 0.025**)**

n.upper **<-** apply**(**n, 2, quantile, 0.975**)**

end.time **<-** Sys.time**()**

time.diff **<-** end.time **-** start.time

## Save outputs

# save.image(file="Base case outputs") # commented out

#-------------------------------------------------------------------------------

# Results

#-------------------------------------------------------------------------------

##Plots

windows**(**record**=TRUE**, 10, 10**)**

par**(**mfrow **=** c**(**3, 1**))**

##Trace plots

plot**(**1**:**n.retain.samples, rep**(**0,n.retain.samples**)**, type**=**"n",

ylim**=**range**(**rmax**)**, xlab**=**"sample", ylab**=**"rmax"**)**

**for(**i **in** 1**:**n.chains**){**

lines**(**1**:**n.retain.samples, res**[[**i**]]$**rmax, col**=**i**)**

**}**

plot**(**1**:**n.retain.samples, rep**(**0,n.retain.samples**)**, type**=**"n",

ylim**=**range**(**n.105**)**, xlab**=**"sample", ylab**=**"n.105"**)**

**for(**i **in** 1**:**n.chains**){**

lines**(**1**:**n.retain.samples, res**[[**i**]]$**n.105, col**=**i**)**

**}**

plot**(**1**:**n.retain.samples, rep**(**0,n.retain.samples**)**, type**=**"n", ylim**=**range**(**K**)**, xlab**=**"sample", ylab**=**"K"**)**

**for(**i **in** 1**:**n.chains**){**

lines**(**1**:**n.retain.samples, res**[[**i**]]$**K, col**=**i**)**

**}**

##Posterior marginals

par**(**mfrow **=** c**(**2, 3**))**

hist**(**rmax**)**; hist**(**n.105**)**; hist**(**K**)**

plot**(**density**(**rmax**))**; plot**(**density**(**n.105**))**; plot**(**density**(**K**))**

##Joint posteriors

par**(**mfrow **=** c**(**1, 1**))**

plot**(**rmax, n.105, main**=**paste0**(**"rmax x n.105 ", "(cor = ", round**(**cor**(**rmax, n.105**)**,4**)**, ")"**))**

plot**(**rmax, K, main**=**paste0**(**"rmax x K ", "(cor = ", round**(**cor**(**rmax, K**)**,4**)**, ")"**))**

##Plot of n

main **=** "Base case model"

##Add data points (N's)

##Log-normal CIs (as in p.77 in Buckland et al. (2001) - Introduction to Distance Sampling)

N2005 **<-** 6251

CV.N2005 **<-** 0.17

point.N **<-** N2005

CV.N **<-** CV.N2005

varian.N **<-** **(**point.N*****CV.N**)^**2

var.log.N **<-** log**(**1**+(**varian.N**/(**point.N**^**2**)))**

C **<-** exp**(**1.96*****sqrt**(**var.log.N**))**

lower.N2005 **<-** N2005**/**C

upper.N2005 **<-** N2005*****C

N2008 **<-** 14264

CV.N2008 **<-** 0.084

point.N **<-** N2008

CV.N **<-** CV.N2008

varian.N **<-** **(**point.N*****CV.N**)^**2 #

var.log.N **<-** log**(**1**+(**varian.N**/(**point.N**^**2**)))**

C **<-** exp**(**1.96*****sqrt**(**var.log.N**))** #

lower.N2008 **<-** N2008**/**C

upper.N2008 **<-** N2008*****C

N2012 **<-** 20389

CV.N2012 **<-** 0.071

point.N **<-** N2012

CV.N **<-** CV.N2012

varian.N **<-** **(**point.N*****CV.N**)^**2

var.log.N **<-** log**(**1**+(**varian.N**/(**point.N**^**2**)))**

C **<-** exp**(**1.96*****sqrt**(**var.log.N**))** #

lower.N2012 **<-** N2012**/**C

upper.N2012 **<-** N2012*****C

##Base case model population trajectory

windows**(**record**=TRUE**, 8,6**)**

par**(**mfrow**=**c**(**1,1**)**, mar **=** c**(**5,5,4,2**)** **+** 0.1, family**=**"serif"**)** # default 'mar = c(5,4,4,2) + 0.1'

main **=** "Base case model"

plot**(**years, n.median, ylim **=** c**(**0, 31000**)**,

type **=** "l", lwd **=** 3, main**=**main, las**=**1, ann**=**F**)**

title**(**ylab **=** "Number of whales (N)", line**=**3.6, cex.lab**=**1.5**)**

title**(**xlab **=** "Year", cex.lab**=**1.5**)**

title**(**main **=** main, cex.main**=**2, line**=-**2**)**

lines**(**years, n.lower, lty **=** 2, lwd **=** 1.5**)**

lines**(**years, n.upper, lty **=** 2, lwd **=** 1.5**)**

lines**(**years, catch, col **=** "grey60", lwd **=** 3**)**

points**(**2005, N2005, pch **=** 16, col **=** "black", cex **=** 2**)**

arrows**(**2005, lower.N2005, 2005, upper.N2005, length**=**0.05, angle**=**90, code**=**3, lwd**=**2**)**

#-------------------------------------------------------------------------------

# END OF CODE

#-------------------------------------------------------------------------------

##CODE FOR IMPLEMENTING THE UPDATED MODEL

##Bayesian population model, MCMC used for fitting model based on “Zerbini et al. (2011) A Bayesian assessment of the conservation status of humpback whales in the WSA”

##Last update 20/Jan/2019 by GAB

##This version sets a prior on number of animals in 2005 and uses backward projection to derive an implied prior for number in the first year of inference (1901)

library**(**parallel**)**

library**(**doParallel**)**

library**(**foreach**)**

library**(**doRNG**)**

#-----------------------------------------------------------------------------

# Function definitions

#-----------------------------------------------------------------------------

pop.trajectory **<-** **function(**rmax, K, catch, z **=** 2.39**){**

##Purpose: Create a population trajectory using the generalized logistic model of Zerbini et al. (2011)

##Inputs:

# rmax - max population growth rate

# K - carrying capacity (population assumed at carrying capacity at time 1)

# catch - vector of catches

# z - exponent in generalized logistic function

##Outputs:

# vector of population sizes, of length equal to length of catch vector

T **<-** length**(**catch**)**

n **<-** numeric**(**T**)**

n**[**1**]** **<-** K

**for(**t **in** 1**:(**T**-**1**)){**

##Equation 1 from Zerbini paper

n**[**t **+** 1**]** **<-** n**[**t**]** **+** n**[**t**]** ***** rmax ***** **(**1 **-** **(**max**(**n**[**t**]**, 0**)** **/** K**)** **^** z**)** **-** catch**[**t**]**

**}**

return**(**n**)**

**}**

diff.k **<-** **function(**K, rmax, n.105, catch**)** **{**

#Purpose: Returns the difference between the projected population trajectory in year 105 (i.e., 2005) given K, rmax and catch and the "known" population value in that year, n.105. This function is called repeatedly by find.k which searches for the value of K that gives a diff closest to 0

n **<-** pop.trajectory**(**rmax, K, catch**)**

diff **<-** n**[**105**]** **-** n.105

return**(**diff**)**

**}**

find.K **<-** **function(**rmax, n.105, catch**){**

##Purpose: Searches for a value of K (population size in year 1) given

# values of rmax, n.105 ("known" number in 2005) and catch. Uses bisection algorithm implemented via uniroot function

tol **<-** 1E**-**3

res **<-** uniroot**(**diff.k, c**(**1, 100000**)**, tol **=** tol, rmax, n.105, catch**)**

#if(res$f.root > tol) stop ("Couldn't find K")

return**(**res**$**root**)**

**}**

lnl **<-** **function(**n**)** **{**

##Purpose: return log-likelihood of pop trajectory n.

# Assumes (1) normal observation of growth rate in 1995-98 and (2) lognormal observation on population size in 2005

##Inputs:

# n - vector of population trajectory, starting in 1901

##Outputs:

# log-likelihood as a scalar

##Log growth rate 1995.1998

robs1995.1998 **<-** 0.074

sigma1995.1998 **<-** 0.033

##Log growth rate 2002.2011

robs2002.2011 **<-** 0.1135

sigma2002.2011 **<-** 0.013

##Population size in 2008 and 2012 (Bortolotto et al. 2017)

N2008 **<-** 14264

CV.N2008 **<-** 0.084

lN2008 **<-** log**(**N2008**)**

sigma.lN2008 **<-** sqrt**(**log**(**1 **+** CV.N2008 **^** 2**))**

N2012 **<-** 20389

CV.N2012 **<-** 0.071

lN2012 **<-** log**(**N2012**)**

sigma.lN2012 **<-** sqrt**(**log**(**1 **+** CV.N2012 **^** 2**))**

##Calculate observed log growth rate

r1995.1998 **<-** **(**log**(**max**(**n**[**98**]**, 0**))** **-** log **(**max**(**n**[**95**]**, 1E**-**7**)))** **/** 3

r2002.2011 **<-** **(**log**(**max**(**n**[**111**]**, 0**))** **-** log **(**max**(**n**[**102**]**, 1E**-**7**)))** **/** 9

#lnl of observed rate

res.1a **<-** **-(**log**(**sigma1995.1998**)** **+** 0.5 ***** **(**r1995.1998 **-** robs1995.1998**)** **^** 2 **/** sigma1995.1998 **^** 2**)**

res.1b **<-** **-(**log**(**sigma2002.2011**)** **+** 0.5 ***** **(**r2002.2011 **-** robs2002.2011**)** **^** 2 **/** sigma2002.2011 **^** 2**)**

#lnl of population size in 2005

res.3 **<-** **-(**log**(**sigma.lN2008**)** **+** lN2008 **+** 0.5 ***** **(**log**(**max**(**n**[**108**]**, 0**))** **-** lN2008**)** **^** 2 **/** sigma.lN2008 **^** 2**)**

res.4 **<-** **-(**log**(**sigma.lN2012**)** **+** lN2012 **+** 0.5 ***** **(**log**(**max**(**n**[**112**]**, 0**))** **-** lN2012**)** **^** 2 **/** sigma.lN2012 **^** 2**)**

res **<-** res.1a **+** res.1b **+** res.3 **+** res.4

**if** **(**is.na**(**res**))** res **<-** **-Inf**

return**(**res**)**

**}**

ln.prior **<-** **function(**rmax, K, n.105**)** **{**

##Purpose: returns log of prior on rmax, K and n in 2005

# Assume (1) uniform prior distribution (0, 0.106) for rmax and

# (2) uniform (500, 22000) prior on n in 2005

##Inputs:

# rmax - value of rmax

# n.105 - population size in 2005

# K - value of K

res.1 **<-** dunif**(**rmax, 0, 0.106, log **=** **TRUE)**

res.2 **<-** dunif**(**n.105, 500, 22000, log **=** **TRUE)**

res **<-** res.1 **+** res.2

**if(**is.na**(**res**))** res **<-** **-Inf**

return**(**res**)**

**}**

generate.samples**<-function(**B, thin, starting.values, catch,

proposal.sd.mult **=** c**(**0.05, 0.05**)**, proposal.rho **=** 0**){**

##Purpose: implements a random walk Metropolis sampler, based on a multivariate normal proposal

##Inputs:

# B - number of samples (before thinning)

# thin - number of samples for each 1 saved

# catch - vector of catches

# starting.values - starting values (list) $rmax and $n.105

# proposal.sd.mult - used for specifying the random walk sd - sd for each parameter (vector length 2)

# is initial value * proposal.sd.mult

# proposal.rho - correlation between proposal for rmax and K

##Outputs:

# rmax - vector of B/thin rmax values

# n.105 - vector of B/thin N2005 values

# K - vector for B/thin K values

# n - matrix (B/thin * length(catch)) abundance values

# p.accept - proportion of proposed values that were accepted

##Implementation note - requires the MASS library

# for multivariate normal distribution

require**(**MASS**)**

##Set up storage of samples

n.to.save **<-** B%/%thin

rmax **<-** n.105 **<-** K **<-** numeric**(**n.to.save**)**

n **<-** matrix**(**0, n.to.save, length**(**catch**))**

##Initialize chain

rmax.current **<-** rmax**[**1**]** **<-** starting.values**$**rmax

n.105.current **<-** n.105**[**1**]** **<-** starting.values**$**n.105

K.current **<-** K**[**1**]** **<-** find.K**(**rmax.current, n.105.current, catch**)**

n.current **<-** n**[**1, **]** **<-** pop.trajectory**(**rmax.current, K.current, catch**)**

ln.post **<-** lnl**(**n.current**)** **+** ln.prior**(**rmax.current, K.current, n.105.current**)**

##Random walk proposal specification

rmax.sigma.prop **<-** rmax.current ***** proposal.sd.mult **[**1**]**

n.105.sigma.prop **<-** n.105.current ***** proposal.sd.mult **[**2**]**

Sigma **<-** matrix**(**c**(**rmax.sigma.prop **^** 2,

rep**(**rmax.sigma.prop ***** n.105.sigma.prop ***** proposal.rho, 2**)**,

n.105.sigma.prop **^** 2**)**, 2, 2**)**

n.accept **<-** 0

**for** **(**i **in** 2**:**B**){**

##Propose new values

prop **<-** mvrnorm**(**1, c**(**rmax.current, n.105.current**)**, Sigma**)**

rmax.prop **<-** prop**[**1**]**

n.105.prop **<-** prop**[**2**]**

##Can't have zero or negative rmax or n.105

**if(**rmax.prop **>** 0 **&** n.105.prop **>** 0**)** **{**

K.prop **<-** find.K**(**rmax.prop, n.105.prop, catch**)**

n.prop **<-** pop.trajectory**(**rmax.prop, K.prop, catch**)**

ln.post.prop **<-** lnl**(**n.prop**)** **+** ln.prior**(**rmax.prop, K.prop, n.105.prop**)**

acceptance.prob **<-** min**(**1, exp**(**ln.post.prop **-** ln.post**))**

accept **<-** runif**(**1, 0, 1**)** **<** acceptance.prob

**if(**accept**)** **{**

n.accept **<-** n.accept **+** 1

rmax.current **<-** rmax.prop

n.105.current **<-** n.105.prop

K.current **<-** K.prop

n.current **<-** n.prop

ln.post **<-** ln.post.prop

**}**

**}**

##Save samples

**if** **((**i**-**1**)** %% thin **==** 0**)** **{**

s **<-** **(**i**-**1**)** %/% thin **+** 1

rmax**[**s**]** **<-** rmax.current

n.105**[**s**]** **<-** n.105.current

K**[**s**]** **<-** K.current

n**[**s, **]** **<-** n.current

**}**

**}**

p.accept **<-** n.accept **/** **(**B **-** 1**)**

return**(**list**(**rmax **=** rmax, n.105 **=** n.105, K **=** K, n **=** n, p.accept **=** p.accept**))**

**}**

#-----------------------------------------------------------------------------

# Inputs

#-----------------------------------------------------------------------------

##Catch data

##Base catches fom Zerbini et al., (2011) Table 4; Note: start in 1901

catch**<-**c**(**0, 0, 0, 180, 288, 240, 1261, 1849, 3391, 6468, 5832, 2881, 999,

1155, 1697, 447, 121, 129, 111, 102, 9, 364, 133, 266, 254, 7, 0,

19, 51, 107, 18, 23, 132, 57, 48, 105, 242, 0, 2, 36, 13, 0, 4, 60,

238, 30, 35, 48, 83, 698, 45, 34, 140, 44, 96, 167, 61, 16, 15, 27,

13, 24, 12, 0, 52, 0, 189, 0, 0, 0, 0, 2, 0, 0, 0, 0, 0, 0, 0, 0, 0,

0, 0, 0, 0, 0, 0, 0, 0, 0, 0, 0, 0, 0, 0, 0, 0, 0, 0, 0, 0, 0, 0, 0, 0**)**

##Project forward to 2040

catch **<-** c**(**catch,numeric**(**35**))**

T **<-** length**(**catch**)**

years **<-** 1**:**T **+** 1900

##MCMC chain specifications

n.chains **<-** 3

B **<-** 5.2E5

thin **<-** 50

n.to.save **<-** B%/%thin

n.saved**<-**n.to.save ***** n.chains

burnin **<-** 4e2

n.retain.samples **<-** n.to.save**-**burnin

##Starting values

starting.values**<-**data.frame**(**

rmax **=** c**(**0.01, 0.07, 0.10**)**,

n.105 **=** c**(**4250, 6250, 8250**))**

#Comment this out to get a different result each time

set.seed**(**1234**)**

start.time **<-** Sys.time**()** #Record time to run

**if(**n.chains**>**1**)** **{**

##Run multiple chains in parallel using foreach construct

cl **<-** makePSOCKcluster**(**n.chains**)**

registerDoParallel**(**cl**)**

res **<-** foreach**(**chain **=** 1**:**n.chains**)** %dorng% **{**

generate.samples**(**B **=** B, thin **=** thin, starting.values **=** starting.values**[**chain,**]**, catch, proposal.sd.mult **=** c**(**0.5, 0.5**))**

**}**

stopCluster**(**cl**)**

**}** **else** **{**

##Run a single chain without using parallelization

res **<-** list**(**generate.samples**(**B **=** B, thin **=** thin, starting.values **=** starting.values**[**1,**]**, catch, proposal.sd.mult **=** c**(**0.5, 0.5**)))**

**}**

##Concatenate results from each chain

**for(**i **in** 1**:**n.chains**){**

res**[[**i**]]$**rmax **<-** res**[[**i**]]$**rmax**[(**burnin**+**1**):**length**(**res**[[**i**]]$**rmax**)]**

res**[[**i**]]$**n.105 **<-** res**[[**i**]]$**n.105**[(**burnin**+**1**):**length**(**res**[[**i**]]$**n.105**)]**

res**[[**i**]]$**K **<-** res**[[**i**]]$**K**[(**burnin**+**1**):**length**(**res**[[**i**]]$**K**)]**

res**[[**i**]]$**n **<-** res**[[**i**]]$**n**[(**burnin**+**1**):**nrow**(**res**[[**i**]]$**n**)**,**]**

**}**

str **<-** "c("

**for(**i **in** 1**:**n.chains**){**

str **<-** paste**(**str, "res[[", i, "]]$rmax", ifelse**(**i **<** n.chains, ", ", ")"**)**, sep**=**""**)**

**}**

rmax**<-**eval**(**parse**(**text**=**str**))**

str **<-** "c("

**for(**i **in** 1**:**n.chains**){**

str **<-** paste**(**str, "res[[", i, "]]$n.105", ifelse**(**i **<** n.chains, ", ", ")"**)**, sep**=**""**)**

**}**

n.105**<-**eval**(**parse**(**text**=**str**))**

str **<-** "c("

**for(**i **in** 1**:**n.chains**){**

str **<-** paste**(**str, "res[[", i, "]]$K", ifelse**(**i **<** n.chains, ", ", ")"**)**, sep**=**""**)**

**}**

K**<-**eval**(**parse**(**text**=**str**))**

str **<-** "rbind("

**for(**i **in** 1**:**n.chains**){**

str **<-** paste**(**str, "res[[", i, "]]$n", ifelse**(**i **<** n.chains, ", ", ")"**)**, sep**=**""**)**

**}**

n**<-**eval**(**parse**(**text**=**str**))**

n.median **<-** apply**(**n, 2, median**)**

n.lower **<-** apply**(**n, 2, quantile, 0.025**)**

n.upper **<-** apply**(**n, 2, quantile, 0.975**)**

end.time **<-** Sys.time**()**

time.diff **<-** end.time **-** start.time

##Save outputs

#save.image(file="Updated_model_outputs")

#-----------------------------------------------------------------------------

# Results

#-----------------------------------------------------------------------------

##Plots

windows**(**record**=TRUE**, 10, 10**)**

##Trace plots

par**(**mfrow **=** c**(**3, 1**))**

plot**(**1**:**n.retain.samples, rep**(**0,n.retain.samples**)**, type**=**"n",

ylim**=**range**(**rmax**)**, xlab**=**"sample", ylab**=**"rmax"**)**

**for(**i **in** 1**:**n.chains**){**

lines**(**1**:**n.retain.samples, res**[[**i**]]$**rmax, col**=**i**)**

**}**

plot**(**1**:**n.retain.samples, rep**(**0,n.retain.samples**)**, type**=**"n",

ylim**=**range**(**n.105**)**, xlab**=**"sample", ylab**=**"n.105"**)**

**for(**i **in** 1**:**n.chains**){**

lines**(**1**:**n.retain.samples, res**[[**i**]]$**n.105, col**=**i**)**

**}**

plot**(**1**:**n.retain.samples, rep**(**0,n.retain.samples**)**, type**=**"n", ylim**=**range**(**K**)**, xlab**=**"sample", ylab**=**"K"**)**

**for(**i **in** 1**:**n.chains**){**

lines**(**1**:**n.retain.samples, res**[[**i**]]$**K, col**=**i**)**

**}**

##Posterior marginals

par**(**mfrow **=** c**(**2, 3**))**

hist**(**rmax**)**; hist**(**n.105**)**; hist**(**K**)**

plot**(**density**(**rmax**))**; plot**(**density**(**n.105**))**; plot**(**density**(**K**))**

##Joint posteriors

par**(**mfrow **=** c**(**1, 1**))**

plot**(**rmax, n.105, main**=**paste0**(**"rmax x n.105 ", "(cor = ", round**(**cor**(**rmax, n.105**)**,4**)**, ")"**))**

plot**(**rmax, K, main**=**paste0**(**"rmax x K ", "(cor = ", round**(**cor**(**rmax, K**)**,4**)**, ")"**))**

##Plot of n

main **=** "Updated model"

##Add data points (N's)

##Log-normal CIs (as in p.77 in Buckland et al. (2001) - Introduction to Distance Sampling)

N2005 **<-** 6251

CV.N2005 **<-** 0.17

point.N **<-** N2005

CV.N **<-** CV.N2005

varian.N **<-** **(**point.N*****CV.N**)^**2

var.log.N **<-** log**(**1**+(**varian.N**/(**point.N**^**2**)))**

C **<-** exp**(**1.96*****sqrt**(**var.log.N**))**

lower.N2005 **<-** N2005**/**C

upper.N2005 **<-** N2005*****C

N2008 **<-** 14264

CV.N2008 **<-** 0.084

point.N **<-** N2008

CV.N **<-** CV.N2008

varian.N **<-** **(**point.N*****CV.N**)^**2 #

var.log.N **<-** log**(**1**+(**varian.N**/(**point.N**^**2**)))**

C **<-** exp**(**1.96*****sqrt**(**var.log.N**))** #

lower.N2008 **<-** N2008**/**C

upper.N2008 **<-** N2008*****C

N2012 **<-** 20389

CV.N2012 **<-** 0.071

point.N **<-** N2012

CV.N **<-** CV.N2012

varian.N **<-** **(**point.N*****CV.N**)^**2

var.log.N **<-** log**(**1**+(**varian.N**/(**point.N**^**2**)))**

C **<-** exp**(**1.96*****sqrt**(**var.log.N**))** #

lower.N2012 **<-** N2012**/**C

upper.N2012 **<-** N2012*****C

#Updated model population trajectory

windows**(**record**=TRUE**, 8,6**)**

par**(**mfrow**=**c**(**1,1**)**, mar **=** c**(**5,5,4,2**)** **+** 0.1, family**=**"serif"**)** # default 'mar = c(5,4,4,2) + 0.1'

main **=** "Updated model"

plot**(**years, n.median, ylim **=** c**(**0, 30000**)**,

type **=** "l", lwd **=** 3, main**=**main, las**=**1, ann**=**F**)**

title**(**ylab **=** "Number of whales (N)", line**=**3.6, cex.lab**=**1.5**)**

title**(**xlab **=** "Year", cex.lab**=**1.5**)**

title**(**main **=** main, cex.main**=**2, line**=-**2**)**

lines**(**years, n.lower, lty **=** 2, lwd **=** 1.5**)**

lines**(**years, n.upper, lty **=** 2, lwd **=** 1.5**)**

lines**(**years, catch, col **=** "grey60", lwd **=** 3**)**

points**(**2008, N2008, pch **=** 16, cex **=** 2, col**=**"red"**)**

arrows**(**2008, lower.N2008, 2008, upper.N2008, length**=**0.05, angle**=**90, code**=**3, lwd**=**2, col**=**"red"**)**

points**(**2012, N2012, pch **=** 16, cex **=** 2, col**=**"blue"**)**

arrows**(**2012, lower.N2012, 2012, upper.N2012, length**=**0.05, angle**=**90, code**=**3, lwd**=**2, col**=**"blue"**)**

#-------------------------------------------------------------------------------

# END OF CODE

#-------------------------------------------------------------------------------
