# Supplementary material for: Alternative method for assessment of southwestern Atlantic humpback whale population status
Source: PLoS One. 2021 Nov 17;16(11):e0259541. doi: 10.1371/journal.pone.0259541 (PMC8598017; doi:10.1371/journal.pone.0259541)
Supplement: S2 Appendix — (DOCX) [file pone.0259541.s002.docx]

## S2 Appendix. Model convergence plots.


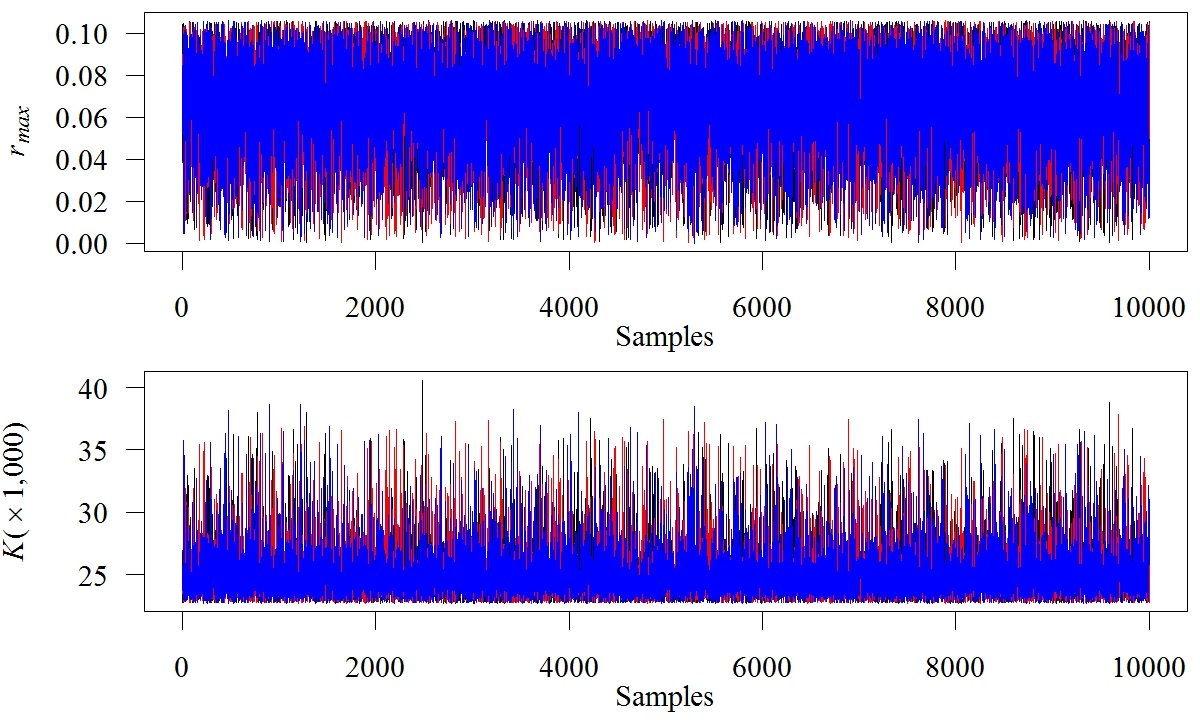


Fig. A. *Base Case model* trace plots for *K* and *r_max_* indicating model convergence, excluding burn-in. Three chains are represented by different colours (black, red and blue).


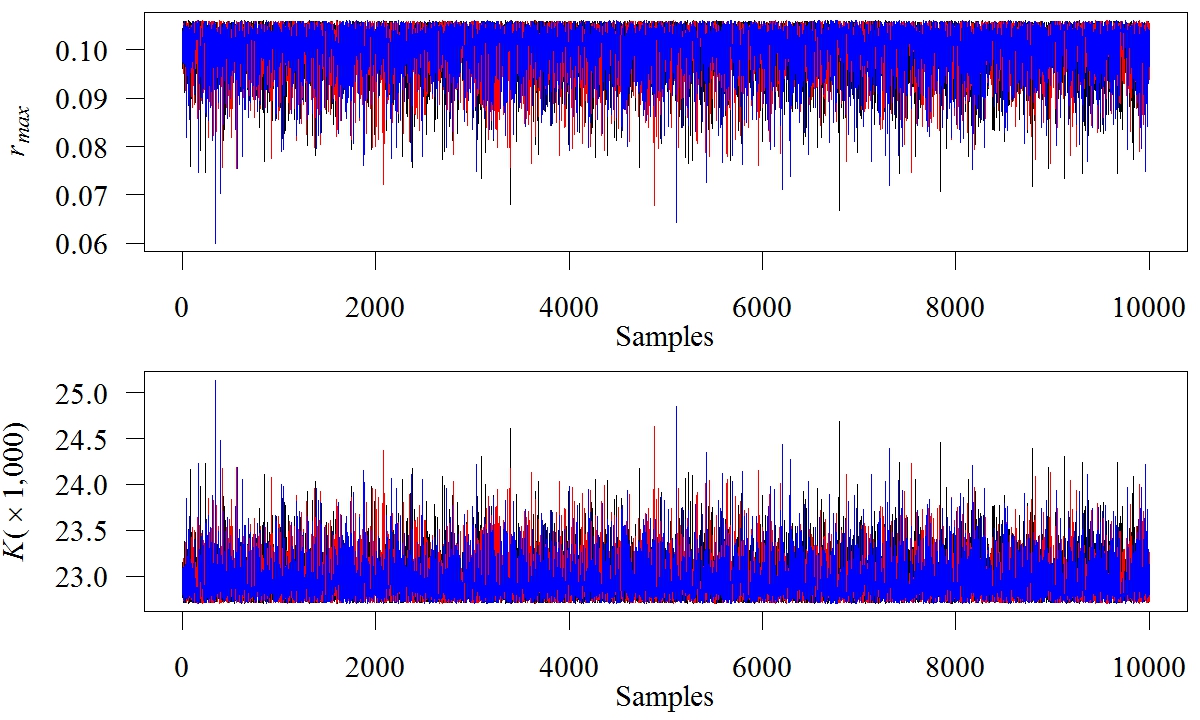


Fig. B. *Updated model* trace plots for *K* and *r_max_* indicating model convergence, excluding burn-in. Three chains are represented by different colours (black, red and blue).
