# Supplementary material for: Alternative method for assessment of southwestern Atlantic humpback whale population status
Source: PLoS One. 2021 Nov 17;16(11):e0259541. doi: 10.1371/journal.pone.0259541 (PMC8598017; doi:10.1371/journal.pone.0259541)
Supplement: S3 Appendix — (DOCX) [file pone.0259541.s003.docx]

## S3 Appendix. Sensitivity analysis plots and table.


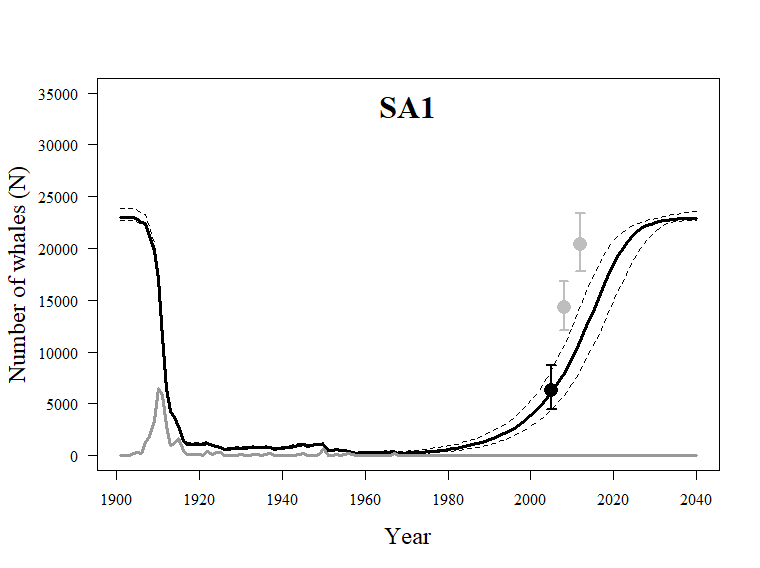

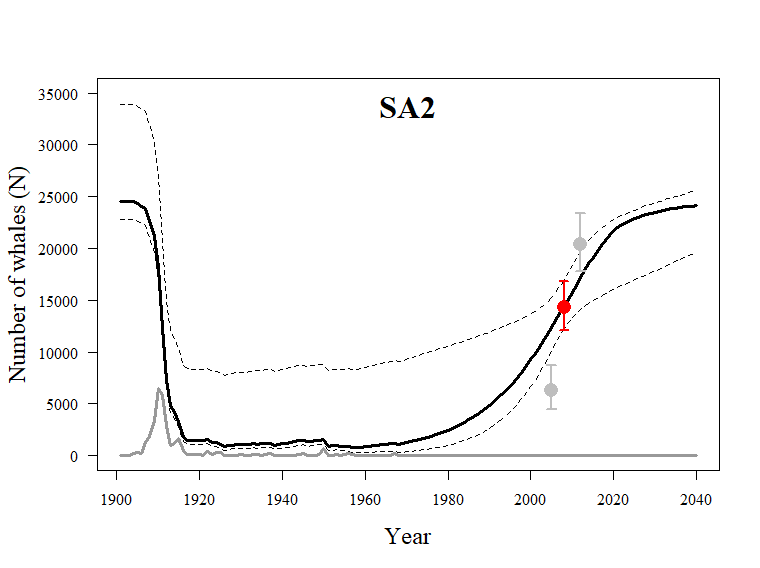


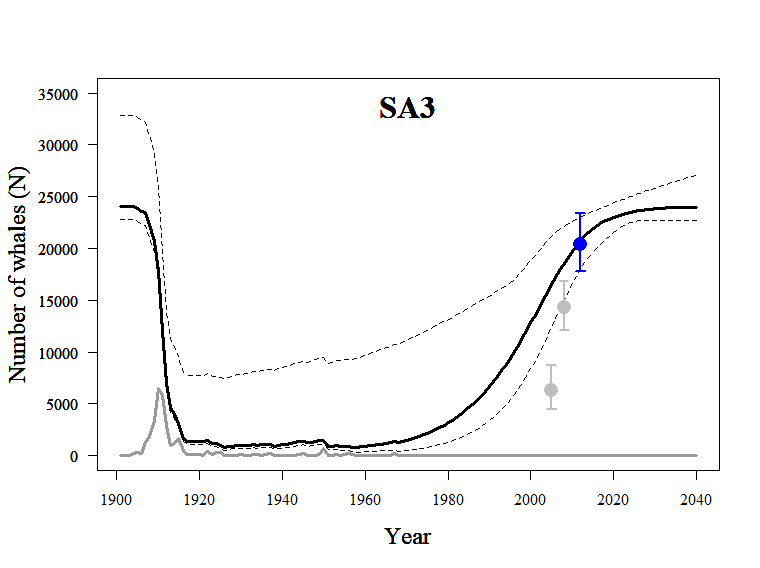

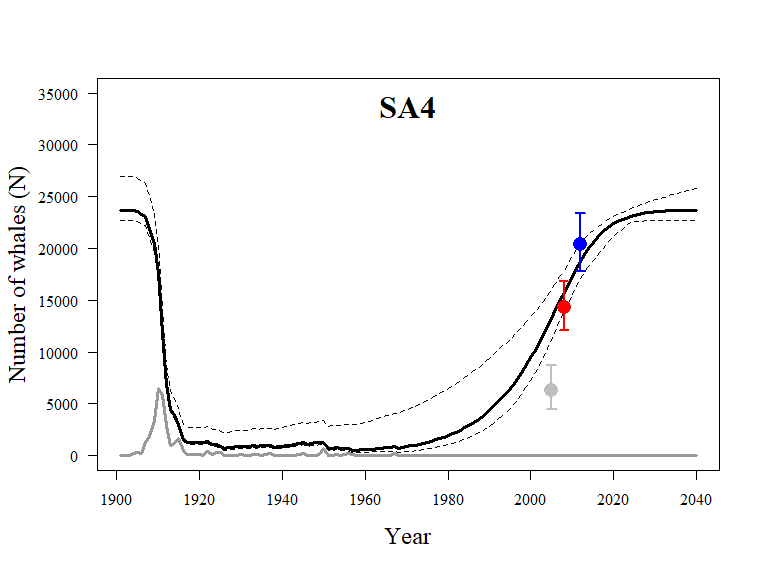


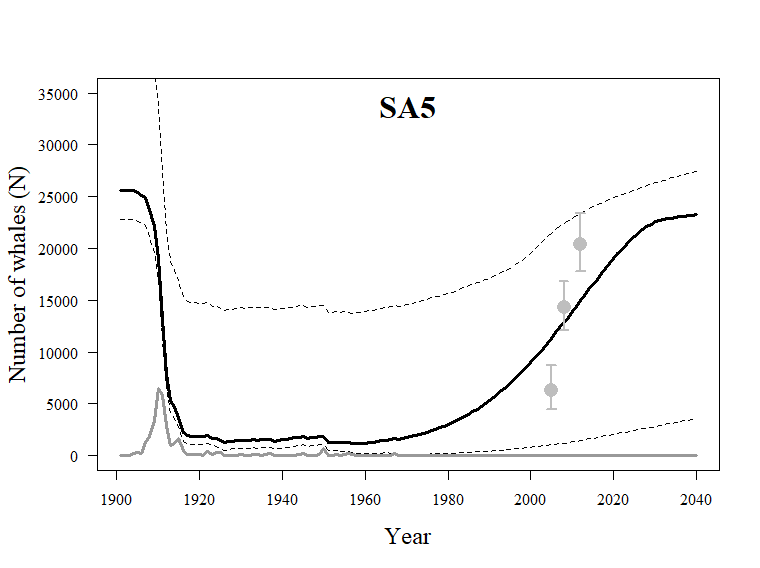

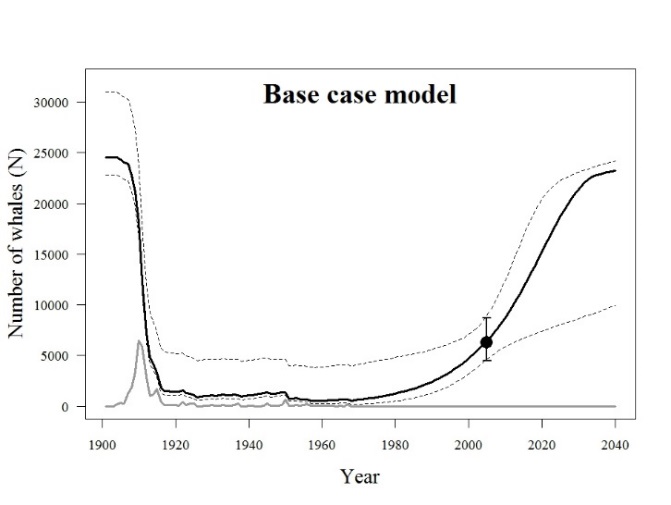


Fig. A. Population trajectory for the five *Sensitivity Analysis* (SA) model scenarios. The *Base Case model* population trajectory is also presented for comparison. Black lines represent the posterior median (solid line) and 95% credible interval limits (dashed lines) estimated for population size. Observed abundances in 2005, 2008 and 2012 are indicated by black, red and blue dots, respectively, with error bars (95% confidence intervals). Grey lines represent catches (*Supporting information 1*). Grey dots with error bars indicate that the abundance data were not used in that SA model. Note the wider scale for the vertical axis in SA2 and SA5 plots. Data inclusion in different SA models are summarized in Table S1.

Fig. B. The Base Case scenario population trajectory from Zerbini et al. (2011) presented in Fig. 3 in that publication.

Table A. Summary of *Sensitivity Analysis* (SA) outputs and data inclusion. Lower and upper 95% credible interval limits are indicated as 2.5% and 97.5%, respectively.

| **SA** | **Parameter** | **Median** | **Mean** | **2.5%** | **97.5%** | **Data** |
| --- | --- | --- | --- | --- | --- | --- |
| **1** | ***r_max_*** | 0.100 | 0.098 | 0.082 | 0.106 | Abundance for 2005,  growth rate for 1995-1998  and growth rate for 2002-2011 |
|  | ***K*** | 22969 | 23052 | 22715 | 23830 |  |
|  | ***N_min_*** | 188 | 202 | 124 | 357 |  |
|  | **Max depletion** | 0.008 | 0.009 | 0.005 | 0.015 |  |
| **2** | ***r_max_*** | 0.070 | 0.067 | 0.012 | 0.104 | Abundance for 2008  and growth rate for 1995-1998 |
|  | ***K*** | 24498 | 25295 | 22795 | 33912 |  |
|  | ***N_min_*** | 769 | 1565 | 289 | 8361 |  |
|  | **Max depletion** | 0.031 | 0.055 | 0.013 | 0.248 |  |
| **3** | ***r_max_*** | 0.078 | 0.074 | 0.018 | 0.105 | Abundance for 2012  and growth rate for 1995-1998 |
|  | ***K*** | 24033 | 24817 | 22764 | 32831 |  |
|  | ***N_min_*** | 784 | 1392 | 529 | 7406 |  |
|  | **Max depletion** | 0.033 | 0.050 | 0.023 | 0.226 |  |
| **4** | ***r_max_*** | 0.085 | 0.081 | 0.040 | 0.105 | Abundance for 2008 and 2012, and growth rate for 1995-1998 |
|  | ***K*** | 23713 | 24006 | 22748 | 26942 |  |
|  | ***N_min_*** | 516 | 789 | 291 | 3022 |  |
|  | **Max depletion** | 0.022 | 0.032 | 0.013 | 0.112 |  |
| **5** | ***r_max_*** | 0.054 | 0.053 | 0.003 | 0.103 | No abundance or growth  rate data |
|  | ***K*** | 25580 | 27136 | 22819 | 40935 |  |
|  | ***N_min_*** | 1123 | 2691 | 257 | 13750 |  |
|  | **Max depletion** | 0.044 | 0.085 | 0.011 | 0.344 |  |
